# Quadriceps arthrogenic muscle inhibition: the effects of experimental knee joint effusion on motor cortex excitability

**DOI:** 10.1186/s13075-014-0502-4

**Published:** 2014-12-10

**Authors:** David Andrew Rice, Peter John McNair, Gwyn Nancy Lewis, Nicola Dalbeth

**Affiliations:** Health and Rehabilitation Research Institute, Auckland University of Technology, Private Bag 92006, Auckland, 1142 New Zealand; Waitemata Pain Services, Department of Anaesthesiology and Perioperative Medicine, North Shore Hospital, 124 Shakespeare Road, Milford, Auckland, 0622 New Zealand; Department of Medicine, University of Auckland, 143 Park Road, Auckland, 1023 New Zealand

## Abstract

**Introduction:**

Marked weakness of the quadriceps muscles is typically observed following injury, surgery or pathology affecting the knee joint. This is partly due to ongoing neural inhibition that prevents the central nervous system from fully activating the quadriceps, a process known as arthrogenic muscle inhibition (AMI). This study aimed to further investigate the mechanisms underlying AMI by exploring the effects of experimental knee joint effusion on quadriceps corticomotor and intracortical excitability.

**Methods:**

Seventeen healthy volunteers participated in this study. Transcranial magnetic stimulation was used to measure quadriceps motor evoked potential area, short-interval intracortical inhibition, intracortical facilitation and cortical silent period duration before and after experimental knee joint effusion. Joint effusion was induced by the intraarticular infusion of dextrose saline into the knee.

**Results:**

There was a significant increase in quadriceps motor evoked potential area following joint infusion, both at rest (*P* = 0.01) and during voluntary muscle contraction (*P* = 0.02). Cortical silent period duration was significantly reduced following joint infusion (*P* = 0.02). There were no changes in short interval intracortical inhibition or intracortical facilitation over time (all *P* > 0.05).

**Conclusions:**

The results of this study provide no evidence for a supraspinal contribution to quadriceps AMI. Paradoxically, but consistent with previous observations in patients with chronic knee joint pathology, quadriceps corticomotor excitability increased after experimental knee joint effusion. The increase in quadriceps corticomotor excitability may be at least partly mediated by a decrease in gamma-aminobutyric acid (GABA)-ergic inhibition within the motor cortex.

## Introduction

Marked weakness and atrophy of the quadriceps muscle is often observed after knee injury, knee surgery or in patients with knee joint arthritis. This is partly caused by an ongoing neural activation deficit of the quadriceps, a process known as arthrogenic muscle inhibition (AMI) (for a review see [1]). The effect of AMI on quadriceps strength is striking, with knee extensor peak torque decreasing by 80 to 90% one to three days after knee joint surgery [2,3]. Despite diminishing over time [4], residual levels of AMI may persist as long as 4 years after initial joint trauma [5]. Furthermore, AMI appears to be ever present in arthritic joint disease [6], accounting for a large amount of the quadriceps muscle weakness observed in these individuals [7].

As well as being a direct cause of quadriceps muscle weakness, AMI may prevent effective muscle strengthening [8,9], leading to long-term quadriceps muscle atrophy and weakness that is difficult to reverse. Ongoing quadriceps weakness is clinically important, as it is associated with impaired dynamic knee-joint stability [10] and physical function [11,12]. Moreover, weaker quadriceps have been associated with an increased rate of loading at the knee joint [13] and recent longitudinal data has shown that greater baseline quadriceps strength may protect against incident knee pain [14], patellofemoral cartilage loss [14] and tibiofemoral joint-space narrowing [15].

Despite its clinical importance, the mechanisms underlying AMI are only partially understood. AMI has been linked to knee joint swelling, pain and structural damage [1]. The relative contribution of these factors is unclear but it is known that swelling alone provokes potent quadriceps AMI. This has been repeatedly demonstrated by the experimental infusion of fluid into healthy, uninjured joints. These studies have shown that intra-articular swelling substantially reduces quadriceps peak torque [16,17], electromyographic (EMG) activity [16,18] and H-reflex amplitude [19,20], despite the absence of structural damage, joint inflammation or pain. It has been reported that as little as 10 ml of fluid may induce inhibition [17] with infusions between 20 and 60 ml capable of reducing maximum effort quadriceps peak torque by 30 to 40% [17,21]. Aspirating or injecting a local anaesthetic into the infused joint largely abolishes AMI [17,20], while the prior injection of local anaesthetic prevents AMI with subsequent joint infusion [17], confirming the role of articular sensory receptors in this process.

Studies in animals [22,23] have shown that joint infusion elevates intra-articular pressure, stimulating stretch and pressure-sensitive mechanoreceptors and greatly increasing group-II joint afferent discharge. Group-II joint afferents are known to excite group-I non-reciprocal (Ib) inhibitory interneurons in the spinal cord [24], which in turn inhibit quadriceps α-motoneurons and prevent full activation of the muscle [25]. Thus, AMI appears to be at least partly mediated by increased spinal reflex inhibition of the quadriceps α-motoneuron pool.

It is unclear whether supraspinal pathways also contribute to AMI. Two studies [26,27] have utilised transcranial magnetic stimulation (TMS) to show that quadriceps corticomotor excitability is altered in individuals with knee joint pathology. However, these cross-sectional studies were performed following long-term joint pathology, making it difficult to elucidate the effects of joint injury from other factors such as disuse, medication use and varying rehabilitation programmes. Furthermore, the magnitude of AMI, and hence its clinical impact, is typically greatest in the acute stages after joint injury or surgery [2-4]. Thus, the purpose of this study was to explore the acute effects of experimental knee joint swelling on quadriceps corticomotor and intracortical excitability. Our hypotheses were that experimental joint infusion would lead to 1) a decrease in quadriceps corticomotor excitability and 2) a reduction in intracortical excitability, reflected by an increase in intracortical inhibition and/or a decrease in intracortical facilitation.

## Methods

### Participants

Seventeen participants (eleven male and six female) volunteered to take part in this study. Participants were screened and excluded based on contraindications to TMS, including epilepsy, head injury, metal implants, or central nervous system-altering medications. Further exclusion criteria were previous history of pathology in both knee joints, a history of lower limb or spinal surgery, or a history of neurological disease. In the event of a previous knee injury, the contralateral (uninjured) knee joint was used in testing. Participants were asked to refrain from ingesting caffeine, alcohol or medication for 4 hours prior to testing. All participants provided written informed consent for the experimental procedures. Ethical approval for this study was granted by the Northern X Regional Ethics Committee, Auckland, New Zealand in accordance with the principles set out in the declaration of Helsinki.

### Participant positioning

Participants were positioned in an isokinetic dynamometer (Biodex 3, Biodex Medical Systems, Shirley, NY, USA) for the duration of the experimental procedures. The lateral epicondyle of the femur was aligned with the dynamometer’s axis of rotation and the knee fixed in 60° of flexion. Straps were firmly secured over the distal tibia, waist and chest to limit extraneous movement during the testing procedures.

### Experimental knee joint infusion

With the knee resting in slight flexion, a 23-g cannula was inserted into the superomedial or, on two occasions, the superolateral aspect of the joint. All injections were performed without local anaesthesia, under strictly sterile conditions. A pressure transducer (Medex Inc., Dublin, OH, USA) and syringe were attached in parallel with the cannula via a three-way tap and pressure-resistant tubing. A dextrose saline solution (4% dextrose and 0.19% NaCl) was injected into the joint space in increments of 15 ml or less. Intra-articular pressure was monitored for each participant and infusion stopped when intra-articular pressure reached 50 mmHg. A standardised pressure of 50 mmHg was chosen as this has previously been shown to lead to notable quadriceps AMI and is well-tolerated by participants [16,18]. Importantly, we chose to standardise the infusion to intra-articular pressure as a set volume of infusion (for example, 60 ml) provides a relatively poor estimate of capsular tension due to large individual differences in both the size of the joint cavity and capsular elastance. Both joint afferent discharge [23] and quadriceps AMI [18] have a stronger correlation with intra-articular pressure compared to intra-articular volume. The pain sensation evoked by the joint infusion was measured after the withdrawal of the cannula from the joint and just prior to the post infusion measurements. Participants were asked to verbally rate any pain they felt in their knee on a scale from 0 (no pain) to 100 (worst pain imaginable).

### Electromyography

To collect quadriceps motor-evoked potentials (MEPs), bipolar Ag-AgCl disc electrodes (Norotrode 20, Myotronics Inc., Kent, WA, USA) with an inter-electrode distance of 2.2 cm were placed on the skin overlying the vastus lateralis muscle belly in accordance with surface electromyography for the non-invasive assessment of muscles (SENIAM) guidelines [28]. A ground electrode (Red Dot, 3 M, St Paul, MN, USA) was positioned slightly below the midpoint of the bony surface of the tibia. Prior to electrode placement the skin was shaved, abraded and cleaned with alcohol to reduce signal impedance. All EMG signals were amplified (x1,000), filtered (10 Hz to 1,000 Hz) (AMT-8, Bortec Biomedical, Alberta, Canada) and sampled at 2,000 Hz (Micro 1401, Cambridge Electronic Design, Cambridge, UK) before being stored on a computer for further analysis. Three 5-s maximum effort voluntary contractions of the quadriceps were performed prior to the first baseline measurements. The largest amplitude root mean square of the vastus lateralis EMG signal within a 1-s window was taken as the MVC. This was used to standardise the level of muscle activation (approximately 10% of MVC) during the TMS measures performed during active muscle contraction.

### Transcranial magnetic stimulation

To minimise the effect of strong voluntary contractions on corticomotor excitability [29], a 5-minute rest period was given between the performance of MVCs and the beginning of TMS procedures. Stimuli were delivered over the scalp using a BiStim 200^2^ and a double cone coil (Magstim Company, Whitland, UK). The coil was placed over the contralateral primary motor cortex so that the induced current flow was in a posterior-anterior direction. First, the optimum site for stimulation (hot spot) was found by delivering a series of suprathreshold stimuli as the coil was systematically moved over the scalp until the largest quadriceps MEP was elicited. This was typically found approximately 1 to 2 cm lateral and anterior to the vertex. The hot spot was marked on the scalp with a felt pen and all further testing completed with the coil held directly over this position. The location of the coil on the participant’s head was checked repeatedly to ensure that the location and angle of stimulation remained unchanged. Resting motor threshold (RMT) was determined using a staircase method and was defined as the lowest stimulation intensity (% of maximum stimulator output) evoking a clearly discernable MEP in four of eight consecutive stimuli. The active motor threshold (AMT) was determined in an identical manner while maintaining a voluntary quadriceps contraction at approximately 10% of MVC. To achieve this level of activation, participants were given real-time visual feedback of their EMG signal.

### Dependent variables

The following dependent variables were examined from the quadriceps: 1) MEP area, 2) cortical silent period (CSP) duration, 3) short interval intracortical inhibition (SICI) and 4) intracortical facilitation (ICF). Single-pulse TMS (test stimuli only) was used to elicit the quadriceps MEPs and the CSP, while paired pulse TMS (conditioning and test stimuli) was used to measure SICI and ICF. MEP area provides a measure of overall corticomotor excitability, while the CSP duration, SICI and ICF provide information on the excitability of intracortical pathways (that is, within the motor cortex itself). MEP area, SICI and ICF were collected with the quadriceps muscle quiescent (resting condition) and during a tonic voluntary muscle contraction at approximately 10% of MVC (active condition). The CSP duration was collected during the active condition only. For the resting condition, the test stimuli were delivered with a stimulation intensity of 120% RMT and the conditioning stimuli at 70% and 90% of RMT for SICI and ICF, respectively. For the active condition, the test stimuli were set to 120% AMT and the conditioning stimuli set to 70% and 90% of AMT for SICI and ICF, respectively. For both the resting and active conditions, an interstimulus interval of 2 ms was used for SICI and 15 ms for ICF.

### Protocol

**Figure 1:**
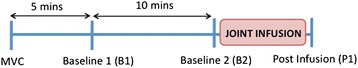
**Diagram illustrating the study protocol.** Maximum effort voluntary contractions (MVC) of the quadriceps were performed prior to the first measurements of the dependent variables. After a 5-minute rest period, transcranial magnetic stimulation was used to measure the dependent variables at baseline 1 (B1), 10 minutes later at baseline 2 (B2) and then immediately after experimental knee joint infusion (P1). At each measurement interval the dependent variables were measured with the quadriceps at rest (resting condition) and then during a quadriceps contraction at 10% of maximum voluntary contraction (active condition).

All TMS parameters were collected at baseline (B1) and again after a 10-minute rest period (B2) (Figure 1). At all measurement intervals, 8 single pulse and 16 paired pulse stimuli (8 SICI, 8 ICF) were delivered first at rest and then during quadriceps contraction. The order of the stimuli was randomised and an interstimulus interval of 6 s was used. Following joint infusion (P1), eight single pulse stimuli were delivered at rest using the same stimulus intensity as B1 and B2. If necessary, the test stimulus intensity was then adjusted for the subsequent measures of SICI and ICF to ensure that a test MEP of the same size (±10%) as B1 test MEP was evoked [30,31]. This process was repeated for the outcome measures in the active condition, with the addition of a further eight single pulse stimuli to assess the CSP. The conditioning stimulus intensity remained unchanged across all measurement points.

### Data processing and analysis

**Figure 2:**
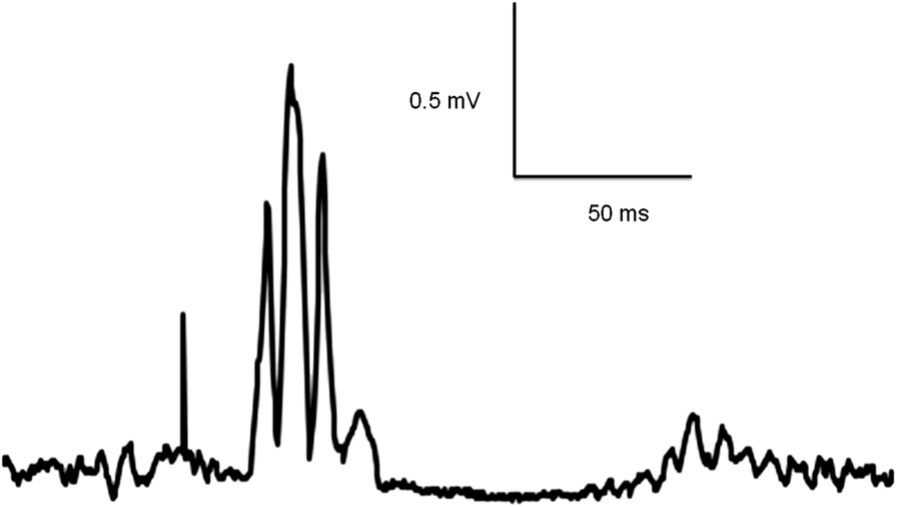
**Rectified motor evoked potential (MEP) recorded from the quadriceps during active muscle contraction.** Note the stimulus artefact (initial large positive deflection) followed by the larger polyphasic MEP and then the silent period in the ongoing EMG following the MEP (cortical silent period).

For the resting condition, the EMG signal was rectified and the 50 ms preceding the stimulus artefact was visually checked for contamination by voluntary muscle activity. Responses were removed from further analysis if silence in the EMG signal was not maintained (<5% of recordings discarded). As the recorded MEPs were typically polyphasic (Figure 2), the area of the averaged, rectified MEP was calculated [32]. For both the resting and active conditions, the MEP areas at the B2 and P1 measurement points were normalised to MEP area at B1. SICI and ICF were determined by expressing the averaged MEP area of the conditioned response relative to the averaged MEP area of the corresponding test (single pulse) response at each measurement period [30]. The CSP duration was calculated as the time from stimulus onset to the first return of the EMG to the mean rectified prestimulus EMG [33].

### Statistical analysis

Normality of the dependent variable distributions was checked using the Shapiro-Wilk test. The one-sample *t*-test was used to assess whether normalised MEP area was significantly different from B1 at the B2 or P1 time points. One-sample repeated-measures analysis of variance (ANOVA) was utilised to analyse differences in SICI and CSP duration over time. In the event that a significant effect of time was found, planned contrasts were used to assess whether the variables differed significantly from B1 at the B2 or P1 time points. As ICF did not conform to a normal distribution in either the resting or active conditions, Friedman’s test was used to analyse this variable over time. Repeated-measures ANOVA was utilised to ensure that the RMS level of background EMG in the 50 ms preceding the stimulus artefact did not differ over time during the active condition. The alpha level for all statistical tests was set to *P* <0.05.

## Results

The mean (±1 SD) age, height and mass of the participants were 32 ± 10 years, 1.78 ± 0.09 metres and 76 ± 14 kg, respectively. Four participants had a history of knee joint pathology in the contralateral (untested) limb. Quadriceps MEPs were unable to be obtained in 3 out of 17 participants during the resting condition (that is, 120% of RMT >100% of maximum stimulator output). Dependent variables were collected from all 17 participants during the active condition. A summary of the main findings is presented in Table 1.

**Table 1:** **Summary of dependent variables at each measurement interval**

	**Baseline 1**	**Baseline 2**	**Post-infusion**
MEP area			
Resting	1.00 ± 0.00	1.05 ± 0.08	1.42 ± 0.13*
Active	1.00 ± 0.00	1.10 ± 0.07	1.30 ± 0.11*
SICI			
Resting	0.38 ± 0.04	0.39 ± 0.04	0.39 ± 0.04
Active	0.75 ± 0.06	0.69 ± 0.04	0.76 ± 0.05
ICF			
Resting	3.10 ± 0.37	3.35 ± 0.53	3.49 ± 0.56
Active	1.61 ± 0.19	1.62 ± 0.22	1.71 ± 0.24
CSP duration (ms)	135 ± 6.06	134 ± 5.97	126 ± 5.93*

Data are presented as mean ± standard error of the mean. *Significant difference from baseline 1 (*P* <0.05). MEP, motor evoked potential area (normalised to baseline 1); SICI, short interval intracortical inhibition (conditioned/unconditioned MEP); ICF, intracortical facilitation (conditioned/unconditioned MEP); CSP, cortical silent period.

### Joint infusion

Upon insertion of the catheter into the knee joint, intraarticular pressure was typically negative or slightly above atmospheric pressure. The median volume required to reach an intra-articular pressure of 50 mmHg was 70 ml (range 15 to 136 ml). Twelve of seventeen participants judged themselves to be completely pain free immediately after the joint infusion procedure; the remainder rated their pain ≤5 out of 100.

### Corticomotor excitability

**Figure 3:**
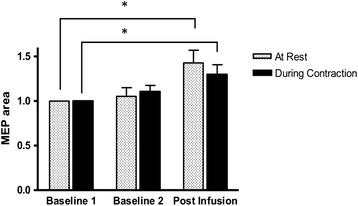
**Quadriceps motor evoked potential (MEP) area (normalised to Baseline 1 MEP area) measured at rest and during voluntary contraction (approximately 10% maximum effort voluntary contraction (MVC)) before and after experimental knee joint infusion.** *Significant difference from baseline 1 (*P* <0.05). Data are mean and one standard error of the mean.

There was no significant difference in quadriceps MEP area between the two baseline measurements in either the resting or the active conditions (both *P* >0.15). Following joint infusion, quadriceps MEP area increased significantly compared to B1 during the resting (*P* = 0.01) and active (*P* = 0.02) conditions (Figure 3). The RMS amplitude of the background EMG during the active condition did not differ across time (*P* = 0.84).

### Intracortical inhibition and facilitation

**Figure 4:**
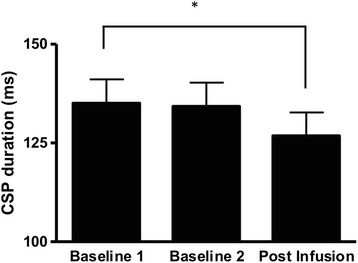
**Quadriceps cortical silent period (CSP) duration before and after experimental knee joint infusion.** *Significant difference from baseline 1 (*P* <0.05). Data are mean and one standard error of the mean.

There was an overall effect of time on CSP duration (*F* = 3.56; *P* = 0.04). CSP duration did not differ between the two baseline measurements (*P* = 0.81). There was a significant decrease in CSP duration following joint swelling (P1) (*P* = 0.02) (Figure 4). There was no significant difference in the magnitude of SICI over time during either the resting or active conditions (both *P* >0.39). There was no significant change in ICF over time for either the resting condition or the active condition (both *P* >0.80). The area of the test MEP used to calculate SICI and ICF did not differ across measurement intervals during either the resting or active conditions (both *P* >0.54).

## Discussion

Knee joint swelling has consistently been shown to prevent full neural activation of the quadriceps muscle [16-18,20,21,34], which is at least partly due to a strong spinal reflex inhibition of the quadriceps α-motoneuron pool [20,25]. Despite these consistent findings across many different studies, we observed a significant increase in quadriceps MEP area after experimental joint infusion, suggesting an increase, rather than a decrease, in quadriceps corticomotor excitability. This observation was made with the muscle at rest and during a voluntary contraction. While somewhat paradoxical, our findings are supported by similar observations in patients with chronic anterior knee pain [27] and anterior cruciate ligament (ACL) injury [26], where quadriceps corticomotor excitability was increased compared to healthy control participants, or the uninjured limb. As corticomotor excitability is partly determined by α-motoneuron excitability, our observations suggest that, despite inhibiting the quadriceps α-motoneuron pool, knee joint swelling leads to an increase in excitability elsewhere in the corticomotor pathway. While speculative, such a change may reflect a compensatory mechanism by the central nervous system in an attempt to maintain neural drive to the muscle.

The current study is the first to use paired pulse TMS to determine the role of intracortical interneurons in the response to articular swelling. We found no evidence that either SICI or ICF are altered by experimental knee joint swelling, suggesting that the excitability of the interneurons underlying these responses was not altered. In contrast, we observed a significant reduction in CSP duration after joint infusion. While the initial 50 to 100 ms of the CSP relates to spinal inhibitory processes, the second component of the CSP (and thus its duration) reflects the suppression of corticospinal output by cortical inhibitory interneurons [35,36]. Furthermore, pharmacological studies have shown that CSP duration is lengthened by gamma-Aminobutyric acid (GABA) re-uptake inhibitors [37] and GABA agonists [38]. Thus, the reduction in CSP duration likely reflects reduced GABAergic inhibition at the level of the motor cortex following joint effusion [39]. Such a change may at least partly explain the observed increase in quadriceps corticomotor excitability.

Interestingly, the observed reduction in CSP duration is at odds with findings in patients who had suffered a unilateral ACL injury, where no side-to-side differences in CSP duration were found despite an increase in quadriceps corticomotor excitability on the injured compared to the uninjured side [26]. This could reflect differences in the mechanism(s) underlying increased corticomotor excitability with acute (experimental) and chronic joint injury. Importantly, other factors relevant to ACL injury such as disuse, pain and a loss of joint afferent input [40] may also influence the excitability of corticomotor and intracortical pathways.

In this regard, it should be noted that while experimental joint infusion provides a model of joint injury that has consistently been shown to induce potent quadriceps AMI, it does not accurately mimic the afferent discharge from a joint affected by trauma or pathology. Experimental joint infusion greatly increases the discharge of group-II joint afferents [23] but in the absence of inflammation, is unlikely to activate a large portion of group-III and -IV afferents. This is demonstrated by the fact that experimental swelling rarely evokes pain, as observed in the current and previous studies [16,34,41]. Thus, it remains to be seen whether an increase in nociceptive output from group-III and -IV knee joint afferents influences corticomotor excitability and/or alters the excitability of intracortical pathways. This should be explored in future research, particularly given recent findings that experimental knee pain impairs quadriceps muscle activation [42] and experimental muscle pain increases intracortical inhibition in hand muscles [43].

## Conclusion

In conclusion, we observed an increase in quadriceps corticomotor excitability following experimental knee joint infusion. This may be at least partly explained by a reduction in cortical GABAergic inhibition, as CSP duration was also reduced. Our findings provide no evidence for a cortical contribution to quadriceps AMI and suggest that ongoing spinal reflex inhibition may be sufficient to explain the marked neural activation deficit that occurs following acute knee injury, knee surgery and in chronic knee joint pathologies. However, further research is needed to confirm these findings in different patient populations and in response to other relevant factors such as joint pain.

## Abbreviations

ACL: anterior cruciate ligament
AMI: arthrogenic muscle inhibition
AMT: active motor threshold
B1: baseline 1
B2: baseline 2
CSP: cortical silent period
EMG: electromyography
GABA: gamma-Aminobutyric acid
ICF: intracortical facilitation
MEP: motor evoked potential
MVC: maximum effort voluntary contraction
RMS: root mean square
RMT: resting motor threshold
SICI: short interval intracortical inhibition
TMS: transcranial magnetic stimulation
